# Antibiotic resistance of *Streptococcus agalactiae* isolated from pregnant women in Garankuwa, South Africa

**DOI:** 10.1186/s13104-015-1328-0

**Published:** 2015-08-20

**Authors:** John Y. Bolukaoto, Charles M. Monyama, Martina O. Chukwu, Sebotse M. Lekala, Maphoshane Nchabeleng, Motlatji R. B. Maloba, Rooyen T. Mavenyengwa, Sogolo L. Lebelo, Sam T. Monokoane, Charles Tshepuwane, Sylvester R. Moyo

**Affiliations:** Department of Life and Consumer Sciences, College of Agriculture and Environmental Science, University of South Africa, Pretoria, South Africa; Department of Microbiological Pathology, University of Limpopo, Medunsa Campus, Pretoria, South Africa; Department of Health Sciences, School of Health and Applied Sciences, Polytechnic of Namibia, P. Bag 13388, Windhoek, Namibia; Department of Obstetrics and Gynaecology, University of Limpopo, Medunsa Campus, Pretoria, South Africa; Department of Medical Microbiology, University of Zimbabwe College of Health Sciences, Harare, Zimbabwe

**Keywords:** Group B streptococcus, Antibiotic resistance, Africa

## Abstract

**Background:**

This study was undertaken to determine the susceptibility profile and the mechanism of antibiotic resistance in Group B streptococcus (GBS) isolates detected in vaginal and rectal swabs from pregnant women attending Dr George Mukhari Academic Hospital, a University Teaching Hospital in Pretoria, South Africa.

**Methods:**

The samples were collected over an 11-month period, cultured on selective media (colistin and nalidixic acid agar and Todd-Hewitt broth), and GBS positively identified by using different morphological and biochemical tests. The susceptibility testing was done using the Kirby–Bauer and E test methods according to CLSI guidelines 2012. The D test method was used for the detection of inducible clindamycin resistance. Multiplex PCR with specific primers was used to detect different genes coding for resistance.

**Results:**

Out of 413 samples collected, 128 (30.9 %) were positive with GBS. The susceptibility testing revealed that 100 % of isolates were sensitive to penicillin, ampicillin, vancomycin and high level gentamicin. Erythromycin and clindamycin resistance was 21.1 and 17.2 %, respectively, in which 69 % had harboured constitutive macrolide, lincosamide and streptogramin B (MLS_B_), 17.4 % had inducible MLS_B_. The M and L phenotypes were present in 6.8 % each. The methylation of target encoded by *ermB* genes was the commonest mechanism of resistance observed in 55 % of isolates, 38 % of isolates had both erm*B* and *linB* genes and efflux pump mediated by *mefA* genes was also distributed among the isolates.

**Conclusions:**

The study reaffirmed the appropriateness of penicillin as the antibiotic of choice for treating GBS infection. However it identified the challenges of resistance to macrolides and lincosamides used as alternative drugs for individuals allergic to penicillin. More GBS treatment options for penicillin allergic patients need to be researched on.

## Background

*Streptococcus agalactiae* (Group B streptococcus, GBS) is the leading cause of neonatal infections in humans. It is an important cause of illness in pregnant women and the elderly with underlying illnesses such as diabetes mellitus or immunosuppression [1–3]. The organism is part of the normal flora of the gut and genital tract and is found colonizing 10–40 % of pregnant women [4].

In adults and pregnant women, GBS can cause urosepsis, chorioamnionitis, endometritis, pneumonia, skin and soft tissue infections [3, 4]. In newborns GBS is the cause of neonatal sepsis, pneumonia, and meningitis [5–7]. Mother to child transmission occurs via the ascending route from the maternal genital tract into the amniotic fluid or at delivery [8]. Infant GBS infection is classified as early-onset disease (EOD) when occurring from birth to 6 days (70–80 % of cases), and late-onset disease (LOD), when it occurs more than 7–90 days after birth; this is transmitted from mother or health care personnel to infants [9–11].

Penicillin and ampicillin are the antibiotics of choice, followed by the first-generation cephalosporins and vancomycin for the treatment of GBS infections [12, 13]. No resistance to penicillin has been reported except a few cases of isolates with intermediate sensitivity or reduced Minimum Inhibitory Concentrations (MICs) to penicillin [14–16]. Alternative antibiotics besides macrolides and lincosamide exist for penicillin allergic patients, although the use of vancomycin should be reserved for penicillin-allergic women with a high risk of anaphylaxis [17–19].

Erythromycin resistance mechanism in GBS is mostly due to ribosomal modification encoded by *erm* genes (*ermB*, *erm A*/*TR*) or through efflux pump mediated by *mefA* genes that cause resistance to 14- and 15-membered macrolides, which confers cross-resistance to all constitutive macrolide, lincosamide and streptogramin B (MLS_B_) antibiotics [20–22]. This resistance can either be inducible (iMLS_B_) or constitutive (cMLS_B_) [23, 24]. Moreover clindamycin resistance in GBS is less frequent and is due to ribosomal translocation encoded by *linB* genes [25]. Multiplex PCR can be used to detect the major erythromycin and clindamycin resistance genes in GBS strains [22, 26].

In South Africa there is a lack of sufficient data on antibiotic resistance in GBS isolates. The purpose of this study was to assess the susceptibility profile of GBS to different antibiotics, to determine genetic basis and the mechanisms of antibiotic resistance in GBS isolates from pregnant women at Dr George Mukhari Academic Hospital, in Pretoria.

## Methods

### Sample collection and culture

The procedure for collection was explained to each patient before specimens were taken. High vaginal swabs (HVS), low vaginal swabs (LVS) and rectal swabs (RS) were collected aseptically from pregnant women (age 18–45 years old); these were at the gestational period of 16–38 weeks, attending antenatal clinic at Dr George Mukhari Academic Hospital, in Garankuwa. This is a University Teaching Hospital associated with the University of Limpopo, Medical University of Southern Africa (Medunsa campus), located about 37 km north of Pretoria, Gauteng Province, in South Africa. It lies at an altitude of about 1350 m (4500 ft) above sea level; in a longitude of 25°37′14″S and latitude of 28°1′1. The women who indicated that they received antibiotic treatment 2 weeks prior to sample collection were excluded. However any women who needed antibiotic treatment after collection were referred for clinical management accordingly. Samples were collected from February 2012 until December 2012. Swabs were placed into Amies transport medium (Rochelle Chemicals and Lab Equipment, Pretoria, South Africa), properly labelled and put into a cooler box containing ice packs, and transported to the laboratory at the Department of Microbiological Pathology, University of Limpopo, Medunsa campus within 2–4 h of collection. Specimens, one per patient were cultured onto selective media, 5 % sheep blood, Columbia colistin and nalidixic acid (CNA) agar (DMP—National Health Laboratory Service, Pretoria, South Africa) and also inoculated into enriched selective GBS broth, Todd-Hewitt broth (DMP—NHLS, Pretoria, South Africa), with the same antibiotics concentration as in CNA (15 mg/l nalidixic acid and 8 mg/l gentamycin) and were incubated for 24–48 h in a 5 % CO_2_ atmosphere at 37 °C. Isolates were confirmed as GBS by using the following methods: morphology of bacteria, haemolytic activity, catalase test, microscopy (Gram’s stain), bile esculin, and CAMP reaction followed by latex agglutination test (Streptex—Slidex ^®^ Strepto Plus—bioMérieux, Marcy l’Etoile, France) for antigen detection.

### Antibiotic susceptibility testing

Purification of isolates was done before susceptibility testing. Susceptibility testing was done on one isolate per patient. For the three methods below, antibiotics were placed onto a Muller-Hinton agar added with 5 % sheep blood following bacterial inoculation (0.5 McFarland of bacterial suspension). The plates were incubated at 37 °C in a CO_2_ enriched environment for 20–24 h. For quality control, *Streptococcus pneumoniae* ATCC 49619 and *Streptococcus agalactiae* ATCC 12403 were used as control strains [16, 27].

#### Disc diffusion

All the 128 GBS positive isolates were tested by Kirby–Bauer method for susceptibility to ampicillin (10 μg), vancomycin (30 μg), high level gentamycin (120 μg), ciprofloxacin (5 μg), chloramphenicol (30 μg), and tetracycline (TE) (30 μg). The results were interpreted according the CLSI 2012 guidelines [27].

#### E test

The MICs of penicillin, erythromycin and clindamycin were determined by commercial paper strips or E test method (AB Biodisk, Davies-Diagnostics, Pretoria, South Africa), following the manufacturer’s instructions.

#### Double disc diffusion

The detection of inducible clindamycin resistance was done by using D test method as previously described [23, 24, 28]. Briefly, erythromycin (15 µg) and clindamycin (2 µg) disks (Oxoid, Davies-Diagnostics, Pretoria, South Africa) were placed 12 mm apart edge to edge [27]. Blunting was defined as growth within the clindamycin zone of inhibition proximal to the erythromycin disk, indicating MLS_B_-inducible methylation. Resistance to both erythromycin and clindamycin indicated MLS_B_-constitutive methylation. Resistance to erythromycin but susceptibility to clindamycin without blunting indicated an efflux mechanism (M phenotype). And finally resistance to clindamycin but susceptible to erythromycin was referred to as L phenotype as previously described [24, 28].

### Molecular techniques

#### DNA extraction

DNA extraction was done using the Zymo Research—DNA MiniPrep-Kit (Zymo-Research—USA, Inqaba Biotechnical Industries, Pretoria, South Africa) and following the manufacturer instructions.

#### Multiplex PCR

All phenotypically resistant GBS isolates were tested to detect three genes for erythromycin resistance, *ermB*, *ermTR*, *mefA* and one gene for clindamycin resistance *linB* using a set of specific primers (Table 1) as previously described [22, 28–30]. The primers were synthesized at Inqaba—Biotechnical industries, Pretoria, South Africa. Briefly, a 50 µl PCR contained 2.5 mM Tris–HCl pH 8.6, 2.5 mM KCl, 2.5 mM MgCl_2_, 5 mM dNTP, 0.5 U *Taq* DNA polymerase (Thermo Scientific—Phusion Flash High-Fidelity PCR Master Mix, AB, Inqaba—Biotechnical Industry, Pretoria, South Africa), PCR water, and 1 µM primers pairs forwards and reverses. A total of 5 µl template DNA was used in the PCR. The cycling conditions on a My Cycler™ thermal cycler (BioRad Laboratories, London, UK) consisted of a single cycle of 95 °C for 3 min followed by 35 cycles of denaturation at 95 °C for 1 min, annealing at 57 °C for 1 min and extension at 72 °C for 1 min. A final extension step of 72 °C for 5 min was followed by a hold at 4 °C as previously described by Desjardins et al. [28].

**Table 1:** PCR primers used for detection of resistance genes in GBS

Gene	Primers	Primers sequence (5′–3′)	Products size (bp)	References
ermB	ermB1	5′_-GAA AAG GTA CTC AAC CAA ATA-3′_(F)	640	[22, 29]
	ermB2	5′_-AGT AAC GGT ACT TAA ATT GTT TAC-3′_(R)		
*ermTR*	ermTR1	5′_-GAA GTT TAG CTT TCC TAA-3′_(F)	400	[22, 28]
	ermTR2	5′_-GCT TCA GCA CCT GTC TTA ATT GAT-3′_(R)		
mefA	mefA1	5′_-AGT ATC ATT AAT CAC TAG TGC-3′_(F)	348	[22, 29]
	mefA2	5′_-TTC TTC TGG TAC TAA AAG TGG-3′_(R)		
linB	linB1	5′_-CCT ACC TAT TGT TTG TGG AA-3′_(F)	944	[22]
	linB2	5′_-ATA ACG TTA CTC TCC TAT TC-3′_(R)		

#### Agarose gel

The different genes of resistance were analyzed based on presence or absence of bands in the agarose gel. Electrophoresis on 1 % agarose gels in 40 mM Tris acetate–2 mM EDTA buffer was used to distinguish PCR products as previously described [4], and bands were visualized using Gel Docs (BioRad Laboratories, London, UK). A culture of GBS ATCC 49447 strain was used as negative control.

### Ethical considerations

The women recruited in the study gave informed and signed consent. The study was approved by the Medical Research and Ethics Committee of South Africa (MREC/P/02/2011: IR) and Directorate for Health and Social Affairs (Medical University of Southern Africa; MEDUNSA) and the College of Agriculture and Environmental Health Sciences, University of South Africa (UNISA).

## Results

Of the 413 pregnant women recruited, 128 (30.9 %) were colonized with GBS in which 70 were recovered from Todd-Hewitt broth and 58 from CNA agar (22/58 RS, 9/58 LVS, 3/58 HVS and 24/58 all the sites). The susceptibility pattern was performed on 128 positive GBS isolates against 9 antimicrobial agents and results are presented in Tables 2 and 3 showing susceptibility profile of GBS isolates by disc diffusion method and by E test, respectively. All strains were 100 % susceptible to penicillin, ampicillin, vancomycin and high level gentamycin. However resistant strains to erythromycin and clindamycin were observed in 21.1 and 17.2 %, respectively. The strains that were resistant to tetracycline were 94.5 % of the isolates, 24.9 % were resistant to chloramphenicol and 18.6 % resistant to ciprofloxacin (all the intermediate values were considered as resistant). The MIC range for penicillin was found to be between 0.012 and 0.12 μg/ml and that for erythromycin and clindamycin both ranged between 0.02 and 0.25 μg/ml. All erythromycin and clindamycin resistant isolates were screened for resistance genes.

**Table 2:** Susceptibility profile of GBS isolates (n = 128)

Antibiotic/method	Susceptible	Intermediate^*^	Resistant
Disc diffusion			
Ampicillin	128 (100 %)	–	–
Vancomycin	128 (100 %)	–	–
Gentamicin-high level	128 (100 %)	–	–
Ciprofloxacin	104 (81.2 %)	17 (13.3 %)	7 (5.5 %)
Chloramphenicol	96 (75.0 %)	11 (8.6 %)	21 (16.4 %)
Tetracycline	7 (5.5 %)	10 (7.8 %)	111 (86.7 %)

* The intermediate values were assimilated to resistant

**Table 3:** Susceptibility profile of GBS isolates by E test (n = 128)

Antibiotic	MIC (µg/ml)				
	Range	50 % of isolates	90 % of isolates	Break point^a^ (susceptible)	% Of isolates resistant
Penicillin	0.002–32	0.012	0.047	≤0.12	–
Erythromycin	0.016–256	0.16	8	≤0.25	21.1
Clindamycin	0.016–256	0.16	8	≤0.25	17.2

^a^CLSI guidelines 2012

The phenotype (by double-disc diffusion) and genotype results of resistant isolates are summarized in Table 4. Among the resistant isolates to both erythromycin and clindamycin, 69 % harboured constitutive MLS_B_, 17.4 % harboured inducible MLS_B_, the M phenotypes were present in 6.8 % and L phenotypes in 6.8 %.

**Table 4:** Minimum inhibitory concentrations of erythromycin and clindamycin resistant isolates, D-shape and screened genes (n = 29)

No	MIC (µg/ml)		D-shape	MLS	Genes
	Erythromycin	Clindamycin			
1	1 (R)	0.50 (I)	Negative	cMLS_B_	*ermB*
2	3 (R)	0.75 (I)	Negative	cMLS_B_	*linB* + *ermB*
3	0.75 (I)	0.75 (I)	Negative	cMLS_B_	*linB* + *ermB*
4	0.50 (I)	0.047 (S)	Negative	M phenotype	*mefA*
5	0.75 (I)	1 (R)	Negative	cMLS_B_	*ermB*
6	4 (R)	0.50 (I)	Negative	cMLS_B_	*ermB*
7	1 (R)	0.38 (I)	Negative	cMLS_B_	*ermB*
8	0.75 (I)	1 (R)	Negative	cMLS_B_	*ermB*
9	0.75 (I)	4 (R)	Negative	cMLS_B_	*linB* + *ermB*
10	4 (R)	7 (R)	Negative	cMLS_B_	*ermB*
11	2 (R)	0.047 (S)	Positive	iMLS_B_	*ermTR*
12	1.5 (R)	0.016 (S)	Positive	iMLS_B_	*ermB*
13	0.75 (I)	0.50 (I)	Negative	cMLS_B_	*linB* + *ermB*
14	8 (R)	0.38 (I)	Negative	cMLS_B_	*ermB*
15	0.25 (S)	2 (R)	Negative	L phenotype	*linB* + *ermB*
16	8 (R)	1 (R)	Negative	cMLS_B_	*linB* + *ermB*
17	0.75 (I)	1 (R)	Negative	cMLS_B_	*ermB*
18	3 (R)	0.38 (I)	Negative	cMLS_B_	*ermB*
19	1 (R)	0.38 (I)	Negative	cMLS_B_	*ermB*
20	1.5 (R)	0.25 (S)	Positive	iMLS_B_	*ermB*
21	0.25 (S)	1 (R)	Negative	L phenotype	*linB* + *ermB*
22	3 (R)	0.047 (S)	Positive	iMLS_B_	*ermB*
23	0.50 (I)	0.75 (I)	Negative	cMLS_B_	*linB* + *ermB*
24	2 (R)	8 (R)	Negative	cMLS_B_	*ermB*
25	0.75 (I)	0.50 (I)	Negative	cMLS_B_	*linB* + *ermB*
26	0.75 (I)	0.094 (S)	Negative	M phenotype	*ermB*
27	4 (R)	0.023 (S)	Positive	iMLS_B_	*ermB*
28	0.50 (I)	1 (R)	Negative	cMLS_B_	*linB* + *ermB*
29	0.75 (I)	0.50 (I)	Negative	cMLS_B_	*linB* + *ermB*

*S* susceptible, *R* resistant, *I* intermediate, *cMLS*_*B*_ MLS_B_-constitutive methylation [erythro (R), clinda (R)], *iMLS*_*B*_ MLS_B_-inducible methylation [erythro (R), clinda (S) with blunting], *M-phenotype* efflux pump mechanism [erythro (R), clinda (S) without blunting], *L-phenotype* erythro (S), clinda (R)

**Fig. 1:**
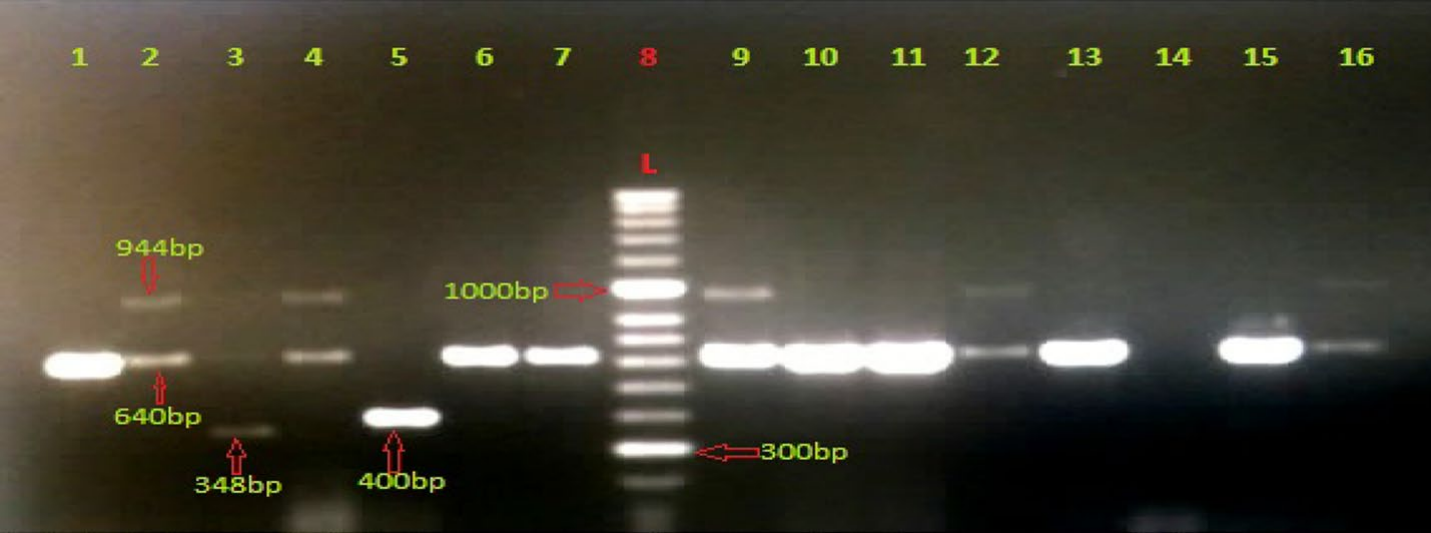
Result of Multiplex PCR of GBS isolates resistant to erythromycin and clindamycin. *Lane 8* DNA Molecular Weight Marker Hyper Ladder™ 50 bp (BioLine). *Lane 14* negative control (GBS ATCC 49447). *Lane 1*, *6*, *7*, *10*, *11*, *13*, *15* presence of *ermB* genes, 640 bp, (sample no 15, 63, 65, 125, 148, 182, 183). *Lane 2*, *4*, *9*, *12*, *16* presence of both *ermB* genes and *linB* genes, 944 bp (sample no 32, 57, 191,159, 184). *Lane 5* presence of *ermTR* gene, 400 bp (sample no 83). *Lane 3* presence of *mefA* gene, 348 bp (sample no 60)

The genotypic analysis of all isolates irrespective of whether they were resistant or sensitive phenotypically, was done using Multiplex PCR. This showed that the methylation of target encoded by *ermB* genes was the commonest mechanism of resistance observed in 55 % (16/29) of isolates, and efflux pump mediated by *mefA* genes were found in 3.4 % of isolates, *ermTR* genes were also found in 3.4 % of isolates and finally both *ermB* and *linB* were observed in 38 % (11/29) of isolates. The PCR products of isolates with resistant genes were distinguished by agarose gel electrophoresis as shown in Fig. 1.

## Discussion

In our study the colonization rate was 30.9 %, the highest reported so far in South Africa. The susceptibility testing was performed on 128 GBS isolated against 9 antimicrobial agents. All strains were 100 % susceptible to penicillin, ampicillin, vancomycin and high level gentamycin. This is similar to a study conducted in Germany and to an Ethiopian study in which 100 % of isolates were found to be sensitive to penicillin, ampicillin, high-level gentamycin and vancomycin [12, 31]. Similar findings were also described in studies from the USA and Argentina where 100 % sensitivity to penicillin, ampicillin and vancomycin [4, 23].

Our findings slightly differ with those described in a study from the neighbouring country of Zimbabwean in which almost 100 % of isolates were sensitive to penicillin, but 2 % were intermediate susceptible to penicillin [8].

In this study 94.5 % of the isolates were resistant to tetracycline, 24.9 % resistant to chloramphenicol, 21.1 % resistant to erythromycin, 18.6 % resistant to ciprofloxacin and 17.2 % resistant to clindamycin. Considering erythromycin and clindamycin resistance rates, similar findings were reported in Canada, in which erythromycin and clindamycin resistant rates were found in 17 and 8 %, respectively [28]. This is again similar to a Tanzanian study that reported a GBS resistance rate of 17.6 and 13 % for erythromycin and clindamycin, respectively and to that described in the Malawian study where the erythromycin resistance rate was 21 % [32, 33]. This suggests that antibiotic resistance in GBS may be similar despite different geographic locations in sub-Saharan Africa. However phylogenetic studies are necessary to verify this. Erythromycin and clindamycin resistance rates in these studies were far lower when compared to the 50.7 % of erythromycin resistance and 38.4 % clindamycin resistance rates reported by Back et al. [18] in the USA, and far lower again than the 54 and 33 %, respectively reported by DiPersio et al. [34]. Erythromycin and clindamycin resistance rates in our findings were higher when compared to the Canadian study, where there was 8 % resistance for erythromycin and 4.5 % for clindamycin [30].

The 94.5 % tetracycline resistance rate found in our study is similar to the 96 % reported by Gray et al. [33], to the 86.8 % reported by De Azavedo et al. [30], and 100 % reported by Moyo et al. [8]. Resistance to tetracycline might be explained by wide and indiscriminate use of these antibiotics worldwide.

In our study, the phenotypic testing by double disk diffusion revealed that 29 isolates were resistant to either erythromycin alone or clindamycin alone or to both erythromycin and clindamycin in which 20 (69 %) isolates harboured cMLS_B_, 5 (17.2 %) harboured iMLS_B_, the M phenotypes were present in 2 (6.8 %) isolates and the L phenotypes in 2 (6.8 %). This finding was in agreement with a Canadian study in which 47.2 % had cMLS_B_ resistance phenotype, 40 % had an iMLS_B_ resistance phenotype, and 12.7 % of the isolates displayed M phenotypes [26]. In Ireland, a study found similar findings with 40 % of isolates that harboured iMLS_B_, 36 % had cMLS_B_, 24 % M phenotype and no L phenotype [24].

Considering the genotypic analysis by multiplex PCR, erythromycin and clindamycin resistance in GBS were mainly associated with *ermB* genes with 55 % of isolates, *ermTR* genes harboured 3.4 % and *mefA* genes 3.4 % of the isolates, both *ermB* and *linB* genes together were present in 38 % of the isolates, and none of the strains carried both *ermB* and *ermTR* nor both *mefA* and *erm* nor *linB* alone. This was similar to a French study in which *ermB* was found in 47 % of isolates, *ermTR* genes in 45 % of isolates and *mefA* gene in 6 % of the isolates and none of the strains carried both *ermB* and *ermTR* or both *mefA* and *erm* genes [17].

In this study, there were two isolates which were phenotypically sensitive to erythromycin, and their *erm* genes were also detected by molecular testing. This may be due to *erm* gene not being expressed, but will require further studies to confirm the interpretation. Also two isolates were resistant to clindamycin but no resistance mechanism was found. This situation could be explained by the fact that isolates may harbour mutations in genes coding for 23S rRNA. A similar situation was also reported in an Irish study where no recognized resistance mechanisms were found in nine isolates [24].

The limitations of the study were that the positive control strain (resistant to both erythromycin and clindamycin) was not available during the molecular stage of the study; thus only negative controls were used. Data from previous local studies in South Africa were not available to allow comparison of genetic mechanisms underlying resistance in GBS.

## Conclusion

This study confirmed the appropriateness of penicillin as still being the antibiotic of choice for treating GBS infections in South Africa. The concern which still remains is the reported increase in the resistance to the macrolides and clindamycin used as alternative drugs for penicillin allergic patients, in other parts of the world. More GBS treatment options for penicillin allergic patients need to be explored. The methylation of targets encoded by *ermB* was the commonest mechanisms of resistance observed and efflux pump mediated by *mefA* genes was also distributed among the isolates. More research studies need to be done in various areas and populations of South Africa to determine GBS colonization.

## Abbreviations

GBS: group B streptococcus
MICs: minimum inhibitory concentrations
cMLS_B_: constitutive macrolide, lincosamide and streptogramin B
iMLS_B_: inducible macrolide, lincosamide and streptogramin B
CNA: Columbia colistin and nalidixic acid agar
